# Preparation of Surfactant-Free Nano Oil Particles in Water Using Ultrasonic System and the Mechanism of Emulsion Stability

**DOI:** 10.3390/nano12091547

**Published:** 2022-05-03

**Authors:** Seon-Ae Hwangbo, Seung-Yul Lee, Bu-An Kim, Chang-Kwon Moon

**Affiliations:** 1 Nanosafety Team, Safety Measurement Institute, Korea Research Institute of Standards and Science (KRISS), 267 Gajeong-ro, Yuseong-gu, Daejeon 34113, Korea; hbsa@kriss.re.kr; 2 The Korea Ship and Offshore Research Institute, Pusan National University, Busan 46241, Korea; sylee7@pusan.ac.kr; 3 Department of Materials Science and Engineering, Pukyong National University, Busan 48513, Korea

**Keywords:** oil-in-water emulsion, surfactant-free emulsification, nano oil particle, emulsion stability, ultrasonic emulsification

## Abstract

Emulsion technology is widely used in the preparation of cosmetics, pharmaceuticals, drug delivery, and other daily necessities, and surfactants are frequently used to prepare these emulsions because of the lack of reliable surfactant-free emulsification techniques. This is disadvantageous because some surfactants pose health hazards, cause environmental pollution, have costly components, and place limitations on process development. In this paper, an efficient method for surfactant-free nano-emulsification is presented. In addition, we discuss the effects of different operating parameters on the oil particle size, as well as the effect of the particle size on the emulsion stability. Specifically, we compared three surfactant-free ultrasonic emulsification technologies (horn, bath, and focused ultrasonic systems). The focused ultrasonic system, which concentrates sound energy at the center of the dispersion system, showed the best performance, producing emulsions with a particle size distribution of 60–400 nm at 400 kHz. In addition, phase separation did not occur despite the lack of surfactants and thickeners, and the emulsion remained stable for seven days. It is expected to be widely used in eco-friendly emulsification processes.

## 1. Introduction

An emulsion is a mixture of two immiscible liquids, in which one is dispersed in the other as small particles. Two representative immiscible liquids are water and oil, which can form different types of emulsions: oil-in-water (O/W), in which oil droplets are dispersed in water, and water-in-oil (W/O), in which water droplets are dispersed in oil. Primary examples of O/W-type food emulsions are milk, ice cream, and mayonnaise, whereas W/O-type foods include butter and margarine. In the emulsification process, surfactants are used to mix these immiscible liquids, and such emulsion technology is employed in various industries and research.

In the pharmaceutical and cosmetic fields, research on small oil particle emulsification (nano-emulsification) technology with uniform particles is being conducted to increase the absorption rate of functional substances and ensure product performance. In addition, the size of the emulsified particles has a great influence on drug delivery, so nano-emulsion preparation is being actively studied [1]. Mixtures of drugs, oils, and surfactants are rapidly dispersed in gastrointestinal fluids after oral administration to form micro- or nano-emulsions containing solubilized drugs [2,3,4].

As discussed, emulsification is typically applied to water and oil mixtures, but the mixing of water and oil without surfactants leads to the rapid separation of each component. Surfactants (emulsifiers) enable the stable mixing of immiscible liquids by changing the interfacial tension of the different phases. Crucially, surfactants prevent phase separation because they contain both lipophilic and hydrophilic groups; thus, the oil-soluble tail projects into the oil, whereas the water-soluble end remains in contact with the water phase. To date, more than 20,000 surfactants have been identified and are used to prepare mixtures of many different immiscible phases [5,6]. However, surfactants can be easily absorbed through the skin, remaining in the heart, liver, lungs, and brain for up to five days. The accumulation of these compounds can cause changes to genes and, thus, cancer and other chronic illnesses [5,6]. In addition, surfactants can denature proteins and other important biochemical components, so frequent exposure to surfactants can alter proteins and damage the skin barrier. Despite their hazards, surfactants are widely used in cosmetics, pharmaceuticals, pesticides, polymers, plastics, textiles, dyes, pigments, paints, and household goods owing to their emulsification, dispersion, wetting, foaming, defoaming, and solubilizing effects. Nevertheless, the health hazards, risk of environmental pollution, production costs, and time required for the research and development of new surfactants are significant drawbacks. Therefore, methods for surfactant-free emulsification are urgently required.

There are two main types of emulsification/dispersion: contact and non-contact. The former method relies primarily on frictional forces to achieve dispersion, and physical methods such as ball milling and jet milling can be used, but the dispersions produced by these techniques can be contaminated by the abraded material produced during grinding, and the energy is insufficient for dispersing nanoparticles. In contrast, ultrasonic dispersion is a non-contact method, and its use in the preparation of emulsions has become a popular research topic [7]. Ultrasonic emulsification uses the energy generated during the repeated growth, contraction, and collapse of bubbles, that is, ultrasonic cavitation [8]. The powerful shock waves generated during cavitation disperse particles into very small droplets [9].

In this study, a surfactant-free nano-emulsification method was developed using an ultrasonic method that eliminates the disadvantages of using surfactants. Three types of ultrasound equipment were compared: bath, horn, and a focused ultrasound system that our group previously developed [10], and the optimized method for emulsification was examined through particle size analyses. The sizes of the oil particles at various frequencies of 180, 270, and 400 kHz were determined using a focused ultrasound system that disperses the nano oil particles. In addition, the effect of the oil particles size generated by each frequency on the emulsion stability was investigated, and the surfactant-free ultrasonic nano-emulsification process was optimized.

## 2. Materials and Methods

### 2.1. Materials

Ultrapure water was used as the dispersion medium (continuous phase), olive oil was used as the dispersed phase, and the emulsion was prepared with 1 wt.% oil. Table 1 lists the details of the dispersion medium and dispersed phase used in the experiments.

### 2.2. Methods

#### Horn, Bath, and Focused Ultrasonic Emulsification

In this study, 1 wt.% olive oil O/W-type emulsions were prepared using three different methods and various operating conditions. The particle size distribution of the emulsions prepared by each method was characterized using a particle size analyzer (LA-960, HORIBA, Kyoto, Japan) to determine which method yielded the best emulsion, i.e., that with the smallest particle size.

**Figure 1:**
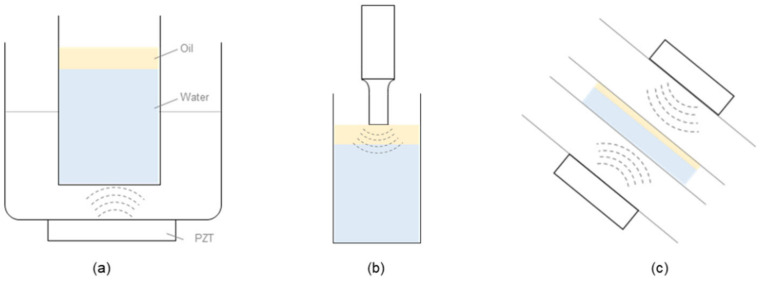
Three methods for ultrasonic emulsification: (**a**) bath-, (**b**) horn-, and (**c**) focused-type ultrasonic systems [10].

Figure 1 shows the three ultrasonic emulsification methods used in this study. In the bath-type method, a bath is filled with a solvent (usually water), and ultrasonic waves are irradiated from the bottom to the top via a piezoelectric element installed at the bottom of the bath. This energy is thereby transmitted from the piezoelectric element at the bottom of the bath to the bath medium (solvent) and then to any immersed medium. The bath-type device used in this work was a JAC-4020 (kodo Technical Research Co., Ltd., Hwaseong, Korea) which has a frequency of 40 kHz and a power of 400 W, equivalent to that of the horn-type device.

In the horn-type ultrasonic method, the time and energy of ultrasonic irradiation are adjusted via a controller, and ultrasonic waves are irradiated from top to bottom via a converter and horn. The horn-type device used in this study was a Sonifier 450 (Marshall Scientific, Manchester, USA), which has a frequency of 20 kHz and power of 400 W.

Both the horn- and bath-type ultrasonic dispersion devices transfer energy in a single direction and, thus, do not uniformly distribute energy through the system. In addition, the heat generated from the devices during operation cannot be controlled, so the emulsification process must be discontinuous. Figure S1 (Supplementary Materials) shows the temperature change of the sample during the ultrasonic operation for the three methods.

To address these drawbacks, we developed a new ultrasonic dispersion method [10]. In this process, the converted acoustic energy is directed toward the center of the system from a cylindrical piezoelectric ceramic element through the surrounding aqueous medium. In addition, the water is circulated at a constant speed to remove the heat generated during operation. Thus, this emulsification method can be used continuously over long periods, making it suitable for commercial use. The performance of the three ultrasonic devices for producing O/W-type emulsions without surfactants or dispersants was compared and evaluated. Table 2 summarizes the experimental conditions used in this study. In the case of the horn-type system, operation for more than 10 min led to heat generation, and the temperature of the dispersion increased above 60 °C. To limit heating, an ice bath was used, and the recommended device operation time of 30 min was selected as the irradiation time.

Ultrasonic energy has a constant amplitude. When an object is irradiated with ultrasonic waves, the object vibrates at the frequency of the applied wave, and the applied energy changes according to the frequency. The size of the oil particles in the water may change according to the change in frequency, and, thus, the stability may change. In this study, the effect of frequency on the preparation of a 1 wt.% olive oil O/W emulsion was investigated. A focused ultrasound system was used in this experiment. Frequencies of 180, 270, and 400 kHz were used, but the power and irradiation time were kept constant. Table 3 lists the operating conditions. The frequency used in the experiment was set to have a difference of approximately 100 kHz or more based on the maximum frequency of 400 kHz because a low frequency was used in the comparative experiment between the bath and the horn. The power was fixed at 100 W, and the effect on frequency was compared. The ultrasonic irradiation time was set to 60 min, and when ultrasonication was performed for a short period of less than 60 min, the phase separation occurred immediately without being completely emulsified, and the particle size did not decrease any longer than 60 min. Therefore, the time was fixed at 60 min.

## 3. Results

### 3.1. Particle Size Distribution

**Figure 2:**
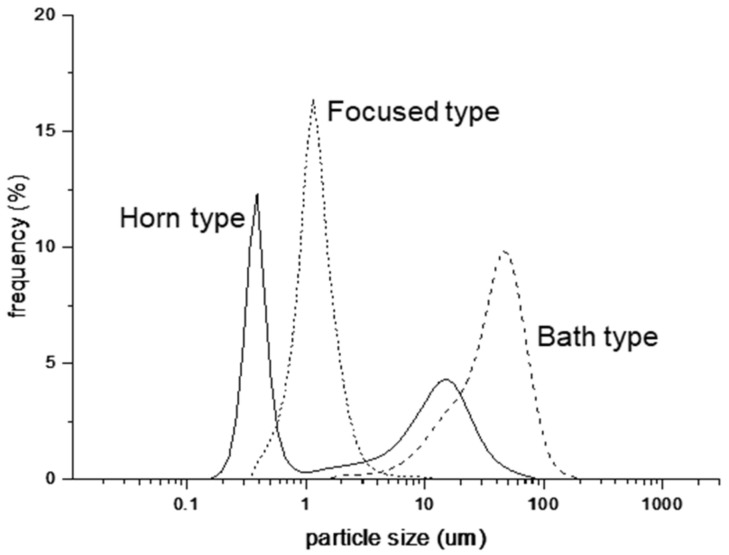
Size distribution of oil particles obtained used horn, bath, and focused methods.

The particle size distribution of the 1 wt.% olive oil O/W-type emulsions differed with the ultrasonic system used (Figure 2).

The emulsions prepared using the horn-type ultrasonic method had a broad particle size distribution ranging from hundreds of nanometers to hundreds of microns. This result suggests that the distribution of energy was not uniform, so the horn-based method was unsuitable for the preparation of emulsions despite the large amount of energy applied to the system. In contrast, the bath-based method yielded oil particles of 2–200 µm. Thus, the bath-based method yields larger particles than the horn-based method, but the particle size distribution is narrower. Crucially, in the horn-based method, ultrasonic waves move from top to bottom, whereas in the bath-based method, ultrasonic waves are transmitted from bottom to top through the medium, thereby delivering insufficient energy for the formation of O/W-type emulsions in both cases. The focused method yielded oil particles of 0.3–8 µm, suggesting more uniformly sized particles than those produced by the horn- and bath-type methods. This finding is likely due to the stronger and more uniform energy transfer in the focused-type method resulting from the cylindrical piezoelectric ceramic element that directly focuses energy on the emulsification medium.

**Figure 3:**
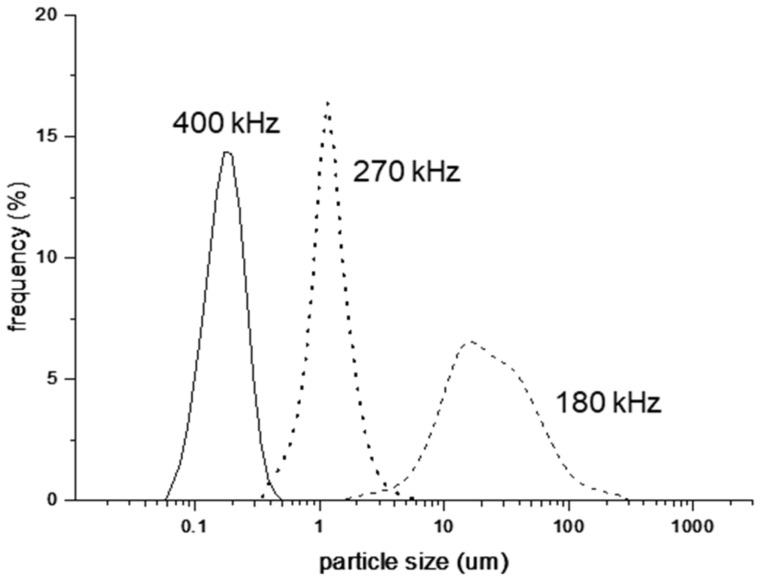
Size distribution of oil particles produced at different frequencies using the focused method.

In summary, the smallest and most uniform particles were produced using the focused method. Therefore, for subsequent experiments, we used the focused method to investigate the correlation between the applied frequency and the size of the oil particles in the O/W emulsions (Figure 3).

### 3.2. Effect of Ultrasonic Frequency on Particle Size

After irradiation at 180 kHz for 200 min, particles of 2–320 µm were formed, which were the least uniform and largest oil particles. In contrast, irradiation at 270 kHz for the same period yielded particles of 0.22–7 µm, substantially smaller and more uniform than those produced at 180 kHz. Further, irradiation at 400 kHz yielded particles of 0.06–0.49 µm in size, which were significantly smaller and more uniform than those produced at other frequencies.

### 3.3. Emulsion Stability

**Figure 4:**
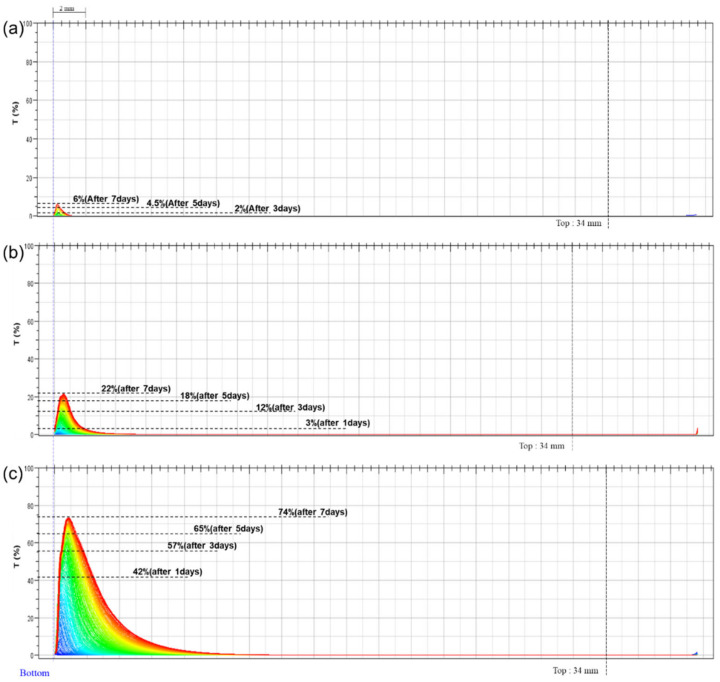
Stability of emulsions prepared at (**a**) 400, (**b**) 270, and (**c**) 180 kHz for 7 d. Color scale: blue to red represents increasing time.

Next, we investigated the stability of these emulsions for 7 d (Figure 4 and Table 4). Figure 4 shows the stability of emulsions prepared using frequencies of 400 kHz (a), 270 kHz (b), and 180 kHz (c), which was analyzed using a Turbiscan (Formulaction, Toulouse, France), and phase separation was confirmed in real time through repeated measurements every 3 h for seven days. Data are presented as the change in transmittance (T, %) with the emulsion height and are color-coded. Here, changing the color from blue to red indicates an increase in time. Table 4 shows the numerical results corresponding to Figure 4. Phase separation according to the time of emulsions A, B, and C was expressed as a percentage (%).

Emulsion A showed a 6% change in transmittance over 7 d, whereas emulsion B showed a 22% change over the same time frame. Emulsion C showed a 42% change after 1 d, indicating rapid emulsion breakdown. Thus, the emulsions with smaller, narrower, and more uniform particle size distributions showed better stability. Further, emulsion B showed more severe breakdown than emulsion A, as reflected in the difference by a factor of 4 or more in the sample height. The change in transmittance was also confirmed to differ by a factor of approximately 4. In summary, emulsion B, which has an overall particle size distribution greater than 1 µm, exhibited a 4-fold reduction in emulsion stability compared to emulsion A, which had a particle size distribution below 1 µm.

## 4. Discussion

### Breakdown and Stabilization Mechanisms of O/W-Type Emulsions

To understand surfactant-free emulsification, the destabilization of O/W-type emulsions must be understood. Phenomena such as aggregation, creaming, and coalescence, which are relevant to the phase separation of O/W-type emulsions, are closely related to particle size. That is, the phase separation of O/W-type emulsions is directly related to the size distribution of the oil particles in the emulsion, and the particle size and its distribution determine the emulsion stability [11,12].

In the case of an O/W-type emulsion, in which water and oil are mixed, particle collapse occurs owing to various physical factors, such as the size of oil particles dispersed in the water phase. These collapse modes can be grouped into three types of emulsions: those having large emulsion particles, small emulsion particles, and small but highly soluble particles. Phase separation occurs via creaming, aggregation, Ostwald ripening, and coalescence [13,14].

Creaming is a result of large differences in the specific gravity of the particles and the dispersion medium. When the viscosity of the dispersion medium is low and the particles are large, creaming is likely. Aggregation involves the mutual attraction of particles by van der Waals forces or electrostatic attraction, thus forming clusters. Owing to the very weak attractive force, aggregates can separate or return to their dispersed state upon the application of weak stimuli/external forces. Ostwald ripening is driven by the surface energy of particles. Specifically, smaller particles in the dispersion system become smaller or disappear, allowing larger particles to grow. Finally, in coalescence, the droplets in the emulsion merge into larger particles. Coalescence is the step immediately preceding two-phase separation into a dispersed phase and a dispersion medium [15].

In O/W emulsions, the sedimentation (or rising) rate of particles increases with increasing particle size or density difference. Based on the viscous resistance and buoyancy that contribute to the creaming separation stability and sedimentation (or rising) speed of particles, there are three ways to control the creaming separation and reduce the settling (rising) velocity of particles. The first method is to reduce the density difference between the dispersed and continuous phases, the second is to increase the viscosity of the continuous phase, and the third is to reduce the size of the particles and distribute them more uniformly. However, density is an inherent property of a substance and cannot be controlled without changing the dispersed phase. Moreover, increasing the viscosity of the continuous phase requires the use of a thickener or other additives, so the viscosity cannot be controlled in additive-free emulsification. The remaining method to prevent creaming and to lower the particle settling velocity is to reduce the oil particle size in the O/W-type emulsion using high-intensity energy. Thus, this method can be used to prepare pure O/W-type emulsions without the use of surfactants or other additives.

In O/W-type emulsions, particles settle (rise) because of density differences, gravity, or viscosity; however, when floating in the liquid, they show random (i.e., Brownian) motion. Specifically, liquid molecules constantly collide with the surfaces of particles suspended in the dispersed phase. These collisions are random and uneven, but they become statistically normalized when the surface of the object is large. Therefore, a large object in a liquid is no longer in motion at hydrostatic equilibrium. However, fine particles on the order of micrometers have small surface areas and experience a large imbalance of collisions, resulting in random motion [13,14]. Hence, particle size affects both the sedimentation velocity and Brownian motion of particles, both of which affect emulsion stability. Specifically, when particles are sufficiently small that their degree of Brownian motion is greater than their sedimentation (rising) velocity, emulsions are physically stabilized without the use of surfactants or other additives [9,10]. The displacement (velocity) of particles with different sizes suspended in water at 20 °C with a density of 0.9 g/cm^3^ and their Brownian motion displacement (velocity) is compared in Table 5 [6,13,14].

As shown in Table 5, the displacement of particles in water is caused by sedimentation and is equal to that from Brownian motion when the diameter of the particle is 1.554 µm. This means that if the particles distributed in the water-based emulsion are 1.554 µm in size (for particles with a density of 0.9 g/cm^3^), the sedimentation and Brownian motion velocities are identical, so the emulsion does not phase-separate or undergoes extremely slow phase separation. Therefore, stable surfactant-free emulsions can be prepared if the particles have a narrow size distribution centered around 1.554 µm. Furthermore, when the particle size is 0.1 µm, the displacement arising from Brownian motion is greater than the sedimentation velocity by a factor of 1000. Thus, phase separation because of Brownian motion is likely to proceed very slowly. Consequently, such emulsions may be stable over periods appropriate for use in commercial products.

## 5. Conclusions

Surfactant-free nano oil particles in water were prepared using bath, horn, and focused dispersion methods at different operating conditions. Surfactant- and dispersant-free emulsions were produced using a variable-frequency focused ultrasonic dispersion device, and the size distribution of the oil particles and emulsion stability over time were characterized. The results revealed that the emulsion with an average particle size below 1 µm (0.06–0.5 µm) produced using the focused-type ultrasonic system at 400 kHz exhibited the best stability. This behavior seems to be the result of the uniform particle size distribution centered below 1.554 µm, which corresponds to the particle size at which the sedimentation displacement of particles becomes equivalent to that due to Brownian motion. The optimal preparation frequency of 400 kHz yielded an emulsion that showed a 6% change in transmittance over seven days, indicating that a stable emulsion had been formed.

Therefore, we expect that our new ultrasonic nano-emulsification method will have applications in a wide variety of fields, such as in pharmaceuticals, drug delivery systems, household goods, and production of eco-friendly cosmetics. Cavitation, which is the main driving force of ultrasonic emulsification, is significantly affected by the surrounding environment. Therefore, further study and optimization of variables such as temperature, pressure, concentration, dissolved gas concentration, and viscosity, which affect cavitation, are required.

## Figures and Tables

**Table 1:** Water and oil used for emulsion preparation.

	Dispersion Medium (Continuous Phase)	Dispersed Phase	O/W-Type Emulsion
Material	3st water (Deionized Water)	Olive oil	-
Specific gravity	1.0	0.915	-
Model or manufacturer	Milli-Q Direct(Merck Millipore, Massachusetts, USA)	F. FAIGES S.L.(Spain)	-
Concentration (wt.%)	-	-	1
Volume (mL)	99	1	100

**Table 2:** Operating conditions for ultrasonic emulsification.

Type	Frequency (kHz)	Power (W)	Irradiation Time (min)	Sample Volume (mL)
Bath type (JAC-4020)	40	400	30	100
Horn type (Sonifier 450)	20	400	30	100
Focused type (developed in our laboratory)	400	100	30	100

**Table 3:** Operating conditions for ultrasonic emulsification at different frequencies.

No.	Dispersion Method	Frequency (kHz)	Power (W)	Irradiation Time (min)
1	Focused	180	100	60
2	Focused	270	100	60
3	Focused	400	100	60

**Table 4:** Stability of emulsions prepared at (emulsion A) 400, (emulsion B) 270, and (emulsion C) 180 kHz over 7 d.

Sample No.	Emulsion A	Emulsion B	Emulsion C
Particle size range	0.06–0.49 µm	0.22–7 µm	2–320 µm
After 1 d	2%	3%	42%
After 3 d	2%	12%	57%
After 5 d	4.5%	18%	65%
After 7 d	6%	22%	74%

**Table 5:** Comparison of the displacement (velocity) of particles of different sizes in water and displacement (velocity) of particles because of Brownian motion.

Sedimentation (Rising) Displacement of Particles (20 °C, Underwater, ρ = 0.9 g/cm^3^)			Particle Displacement by Brownian Motion (20 °C, Underwater)	
Particle Size (µm)	Displacement after 1 s (µm)		Particle Size (µm)	Displacement after 1 s (µm)
0.01	2.18 × 10^−5^	<	0.01	6.55
0.1	2.18 × 10^−3^	<	0.1	2.07
0.35	2.67 × 10^−2^	<	0.35	1.11
0.5	5.44 × 10^−2^	<	0.5	0.93
1	2.18 × 10^−1^	<	1	6.55 × 10^−3^
1.554	5.26 × 10^−1^	=	1.554	5.26 × 10^−1^
3.5	2.67	>	3.5	3.50 × 10^−1^

## Data Availability

The data presented in this study are available on request from the corresponding author.

## Supplementary Materials

The following supporting information can be downloaded at: https://www.mdpi.com/article/10.3390/nano12091547/s1, Figure S1. The temperature change of Samples according to ultrasonic irradiation time (Focused, Bath, Horn ultrasonification).
