# Supplementary material for: Preparation of Surfactant-Free Nano Oil Particles in Water Using Ultrasonic System and the Mechanism of Emulsion Stability
Source: Nanomaterials (Basel). 2022 May 3;12(9):1547. doi: 10.3390/nano12091547 (PMC9101067; doi:10.3390/nano12091547)
Supplement: Supplementary file 1 [file nanomaterials-12-01547-s001.zip › nanomaterials-1656161-supplementary.pdf]

Supplementary Material

Preparation of Surfactant-Free Nano Oil Particles in Water Using Ultrasonic System and the Mechanism of Emulsion Stability

Seon-Ae Hwangbo [1], Seung-Yul Lee [2], Bu-An Kim [3,*] and Chang-Kwon Moon [3,*]

1 Nanosafety Team, Safety Measurement Institute, Korea Research Institute of Standards and Science (KRISS), 267 Gajeong-ro, Yuseong-gu, Daejeon 34113, Korea; hbsa@kriss.re.kr
2 The Korea Ship and Offshore Research Institute, Pusan National University, Busan 46241, Korea; sylee7@pusan.ac.kr
3 Department of Materials Science and Engineering, Pukyong National University, Busan 48513, Korea
* Correspondence: kimba@pknu.ac.kr (B.-A.K.); moonck@pknu.ac.kr (C.-K.M.); Tel.: +82-051-629-6356(B.-A.K. & C.-K.M.)

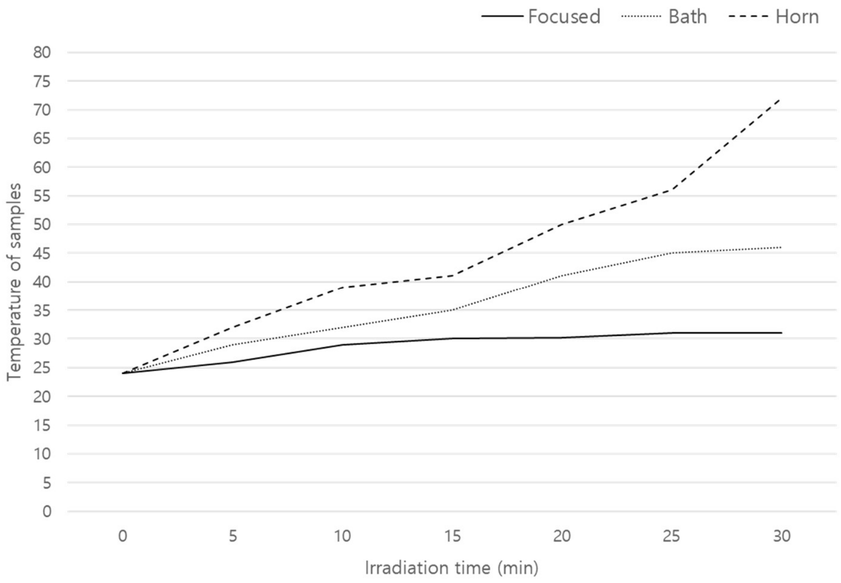

Figure S1. The temperature change of Samples according to ultrasonic irradiation time (Focused, Bath, Horn ultrasonification)
